# The Ser/Thr Kinase PknH Is Essential for Maintaining Heterocyst Pattern in the Cyanobacterium *Anabaena* sp. Strain PCC 7120

**DOI:** 10.3390/life8030034

**Published:** 2018-08-24

**Authors:** Shun-ichi Fukushima, Shigeki Ehira

**Affiliations:** Department of Biological Sciences, Graduate School of Science, Tokyo Metropolitan University, 1-1 Minami-Osawa, Hachioji, Tokyo 192-0397, Japan; fukushimash1986@gmail.com

**Keywords:** pattern formation, cyanobacteria, heterocyst, serine/threonine kinase, time-lapse analysis

## Abstract

In the filamentous cyanobacterium *Anabaena* sp. strain, PCC 7120, heterocysts (which are nitrogen-fixing cells) are formed in the absence of combined nitrogen in the medium. Heterocysts are separated from one another by 10 to 15 vegetative cells along the filaments, which consist of a few hundred of cells. *hetR* is necessary for heterocyst differentiation; and *patS* and *hetN*, expressed in heterocysts, play important roles in heterocyst pattern formation by laterally inhibiting the expression of *hetR* in adjacent cells. The results of this study indicated that *pknH*, which encodes a Ser/Thr kinase, was also involved in heterocyst pattern formation. In the *pknH* mutant, the heterocyst pattern was normal within 24 h after nitrogen deprivation, but multiple contiguous heterocysts were formed from 24 to 48 h. A time-lapse analysis of reporter strains harboring a fusion between *gfp* and the *hetR* promoter indicated that *pknH* was required to suppress *hetR* expression in cells adjacent to the preexisting heterocysts. These results indicated that *pknH* was necessary for the lateral inhibition of heterocyst differentiation to maintain the heterocyst pattern.

## 1. Introduction

*Anabaena* (*Nostoc*) sp. strain PCC 7120 (hereafter *Anabaena*) of the phylum *Cyanobacteria* is a model organism for observing prokaryotic cellular differentiation. *Anabaena* is a filamentous cyanobacterium, that forms unbranched multicellular filaments. In the presence of combined nitrogen, the filaments consist of only vegetative cells, which perform oxygenic photosynthesis. After depriving the cells of combined nitrogen, specific vegetative cells differentiate into heterocysts, which are cells that are specialized for nitrogen fixation, with a semiregular spacing of one heterocyst surrounded by approximately 10 vegetative cells [1,2]. When the vegetative cells between the heterocysts proliferate and increase, one vegetative cell in the middle of a string of vegetative cells differentiates into a heterocyst to maintain the spatial pattern.

The HetR protein is a master regulator of heterocyst differentiation and is necessary and sufficient for this process [3,4]. In response to nitrogen step-down, *hetR* expression increases in specific vegetative cells, and then the HetR-induced cells differentiate into heterocysts [5]. *patS* is induced at the early stages of heterocyst differentiation and plays a key role in heterocyst pattern formation [6,7]. The *patS* mutant exhibits the phenotype of multiple contiguous heterocysts (Mchs). The C-terminal pentapeptide or hexapeptide of PatS ([E]RGSGR) binds to HetR and inhibits its DNA-binding activity [8,9,10]. The RGSGR-containing peptides are transferred from the proheterocysts to adjacent vegetative cells, where heterocyst differentiation is suppressed [11]. The Mch phenotype is also observed in the mutants of the *hetN* gene, which is highly expressed in heterocysts at the late stages of differentiation [12,13]. The RGSGR motif is located in the central part of HetN and is necessary for the suppression of the Mch phenotype [14,15]. In the *hetN* mutant, the initial spatial pattern of the heterocysts is normal, but the Mch phenotype is observed after a prolonged incubation period under diazotrophic growth conditions. Therefore, PatS is involved in initial pattern formation after removal of combined nitrogen, and HetN is required for maintenance of the heterocyst pattern during diazotrophic growth.

*pknH*, which encodes a Ser/Thr kinase, is exclusively expressed in the heterocysts [16]. In a previous report, it was proposed that *pknH* is involved in stabilizing cell junctions, particularly those between heterocysts and vegetative cells [16]. The *pknH* mutant can form heterocysts with nitrogenase activity. However, most heterocysts are detached from the filaments, resulting in filament fragmentation. Thus, the *pknH* mutant exhibits a growth defect under diazotrophic growth conditions. In the present study, we found that the *pknH* mutant exhibits the Mch phenotype. At 24 h after nitrogen deprivation, the heterocyst pattern of the *pknH* mutant was normal, but contiguous heterocysts were formed during the subsequent 24 h. We conducted time-lapse analysis of heterocyst development from 24 to 48 h after nitrogen deprivation and found that the vegetative cells adjacent to the preexisting heterocysts differentiated into heterocysts, resulting in the Mch phenotype. We also report here the dynamics of *hetR* expression in the heterocysts’ neighboring cells in the wild-type (WT) strain and the *pknH* mutant.

## 2. Materials and Methods

### 2.1. Bacterial Strains and Culture Conditions

*Anabaena* and its derivatives were grown in BG-11 medium (containing NaNO_3_ as a nitrogen source), as previously described [17]. Liquid cultures were infused with air containing 1.0% (*v*/*v*) CO_2_. For nitrogen-deprivation experiments, cells grown in BG-11 medium up to the mid-logarithmic phase (OD_750_ of 0.4–0.5) were washed three times with nitrogen-free medium (BG-11_0_) and then resuspended in BG-11_0_ medium containing 5 mM NaHCO_3_. Spectinomycin or neomycin was added to the medium at a final concentration of 10 or 30 µg mL^−1^, respectively, as required.

### 2.2. Construction of Mutant Strains

To construct complementation strains, after substituting Asn for Asp at position 184, *pknH* and *pknHD184N* were inserted into its original locus on the chromosome of the *pknH* disruptant DR1336S [16], as follows. A DNA fragment containing the *pknH* gene was amplified by PCR using the primer pair 1336-5F and PknH-R (Table 1) and cloned between the *SacI* and *SalI* sites of the suicide vector pSU101 [18] to construct pSpknH. The plasmid pSpknHD184N, which contains the *pknHD184N* allele, was generated by site-directed mutagenesis using the PrimeSTAR Mutagenesis Basal Kit (TaKaRa Bio, Inc., Otsu, Japan) and pSpknH as templates. The resultant plasmids were transferred into DR1336S according to the method of Elhai et al. [19], and single recombinants were selected on a BG-11 plate containing spectinomycin and neomycin.

To construct green fluorescent protein (GFP) reporter strains, the plasmid pRhetRG [5], which harbors a transcriptional-fusion of *gfp* and the *hetR* promoter, was transferred into the WT strain and DR1336G, and single recombinants were selected.

### 2.3. RNA Analysis

Total RNA was purified from whole filaments according to the method of Pinto et al. [20], and residual genomic DNA was removed by treating with DNase I (TaKaRa Bio, Inc., Shiga, Japan). Quantitative reverse transcription qRT-PCR was performed as previously described [21] using the primer pairs listed in Table 1.

### 2.4. Microscopic Analysis of Development of Heterocyst Spatial Patterns

Filaments of *Anabaena* suspended in 5 µL of BG-11_0_ medium were applied to a thin (0.5 mm) pad of BG-11_0_ medium containing 1% agarose on a chambered cover glass (AGC Techno Glass, Shizuoka, Japan) prepared by following the method described by Aldea et al. [22]. The filaments on the agarose pad were incubated at 30 °C with continuous illumination at 30 µmol photons m^−2^ s^−1^, and fluorescent images of GFPs and phycobilisomes were taken using the AXIO Imager A2 fluorescence microscope (Carl Zeiss AG, Oberkochen, Germany) with high-efficiency filter sets 38 and 50, respectively. Small black dots were painted on the cover glass as positional markers to track the same microscopic field during sequential observations. The images were captured with a DP73 digital camera (Olympus Corporation, Tokyo, Japan). To quantify the fluorescence intensity, images were converted to grayscale, and the noise was removed using a median filter (5 by 5 cross-shaped window). The fluorescence intensity of each cell was measured using ImageJ v1.46r (National Institutes of Health, Bethesda, MD, USA). GFP fluorescence levels for each cell were normalized using the following formula:

$$\;nv_{i}(t)\;=\frac{v_{i}\left(t\right)}{\left(v_{+5}\left(t\right)+v_{-5,t}\left(t\right)\right)*0.5}$$

where $v_{i}\left(t\right)$ is the intensity value in cell position *i* at time *t*, and *nv* is the normalized GFP fluorescence level.

## 3. Results

### 3.1. Spatial Pattern of Heterocysts in the pknH Mutant

**Figure 1:**
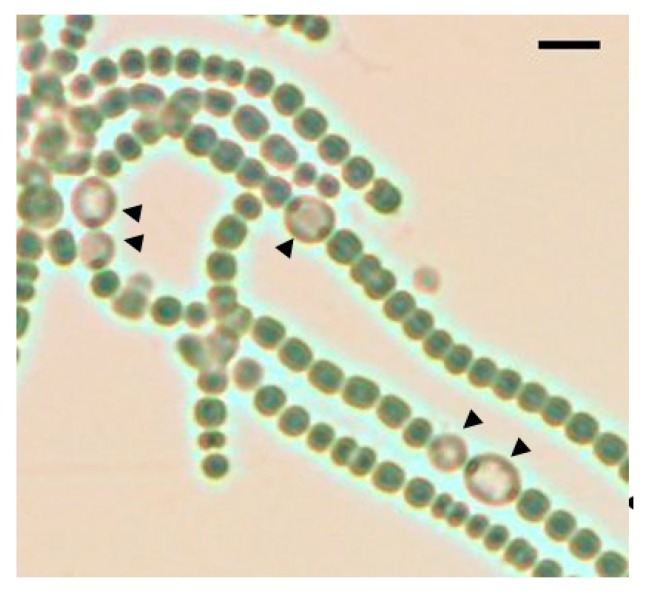
A bright field image of the *pknH* mutant after 48 h of nitrogen deprivation. Black arrowheads indicate heterocysts. Scale bar, 10 μm.

**Figure 2:**
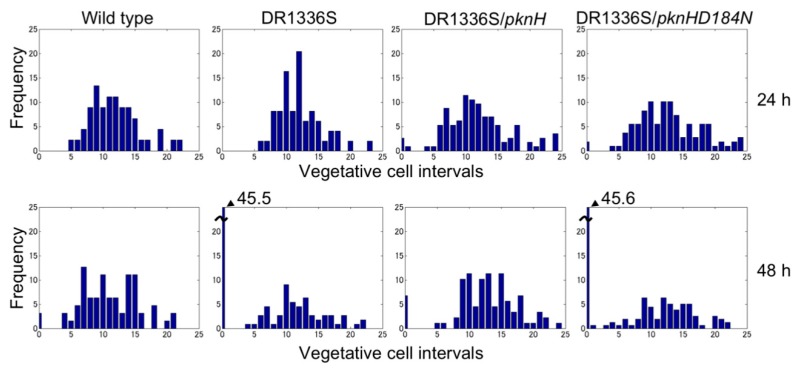
Heterocyst distribution in the wild type, the *pknH* mutant (DR1336S), DR1336S complemented with *pknH* (DR1336S/*pknH*), and DR1336S complemented with the *pknHD184N* allele (DR1336S/*pknHD184N*). Filaments of each strain grown in the presence of nitrate were shifted to nitrogen-free medium and incubated for 24 or 48 h. More than 500 vegetative cells were counted for each sample.

**Figure 3:**
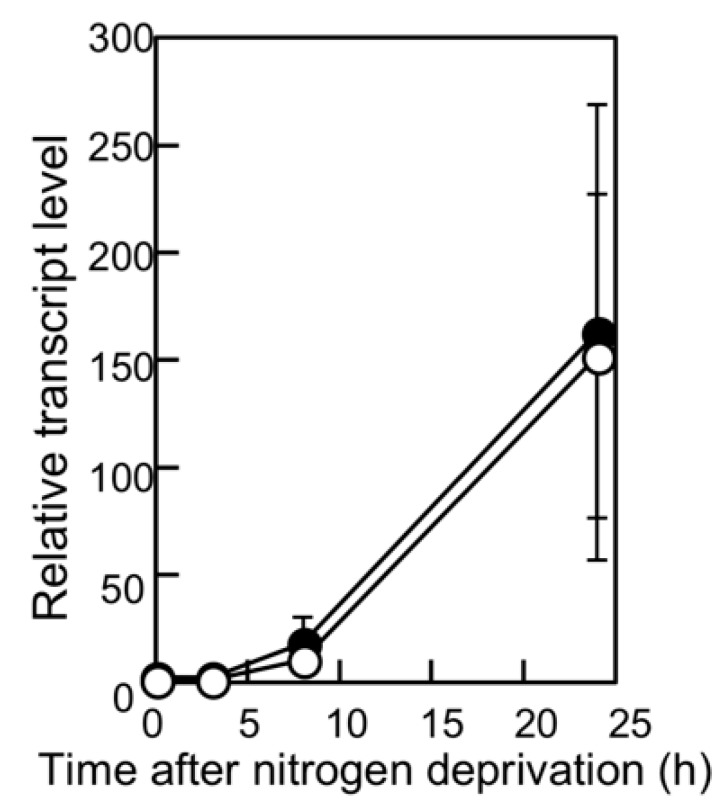
Changes in the *hetN* transcript level after nitrogen deprivation. The relative transcript levels of *hetN* were determined by qRT-PCR in the wild-type (WT) strain (open circles) and the *pknH* mutant (closed circles). RNA samples were prepared from three independently grown cultures. The transcript level at 0 h of the WT strain was designated as 1.

We have previously reported that filaments in the *pknH*-deleted mutant were fragmented during incubation in the absence of combined nitrogen, resulting in many heterocysts being detached from the filaments. Hence, we proposed that *pknH* is involved in maintaining connections between heterocysts and vegetative cells [16]. We attempted to maintain the connections between the heterocysts and filaments in the *pknH* mutant. To prevent the heterocyst detachment that is caused by bubbling with air containing 1% (*w*/*w*) CO_2_, heterocyst differentiation was induced in the nitrogen-free medium containing 5 mM NaHCO_3_ with neither aeration nor agitation. We found pairs of heterocysts and, at times, three or four contiguous heterocysts in the filaments of the *pknH* mutant (Figure 1). Figure 2 shows the number and distribution of the vegetative cells separating two heterocysts. After 24 h of nitrogen deprivation, most heterocysts were separated by 8–15 vegetative cells, and few contiguous heterocysts were formed in either the WT strain or the *pknH* mutant. However, after 48 h, the percentage of contiguous heterocysts was greatly increased in the *pknH* mutant as compared to the WT strain. Therefore, the Mch phenotype in the *pknH* mutant appeared from 24 to 48 h after nitrogen deprivation. Because the phenotype of the *pknH* mutant closely resembles that of the *hetN* mutant [13], the expression of *hetN* after nitrogen deprivation in the *pknH* mutant was determined (Figure 3). Changes in the transcript level of *hetN* in response to nitrogen deprivation were comparable in the *pknH* mutant and the WT strain.

The PknH protein has a Ser/Thr protein kinase domain with 12 amino acid residues that are conserved among the catalytic domains of Ser/Thr kinases. An Asp residue at position 184 of PknH corresponds to the Asp residue of the highly conserved Asp-Phe-Gly motif of the activation loop, which is necessary for phosphorylation activity [23,24]. A *pknH* gene encoding a PknH protein with a substitution of Asn for Asp at position 184 (*pknHD184N*) was introduced into the genome of the *pknH* mutant. Although the heterocyst pattern was recovered by complementation with the original *pknH*, the strain having *pknHD184N* showed a Mch phenotype that was similar to that of the *pknH* mutant (Figure 2), which indicated that Asp-184 of PknH is necessary for suppression of the Mch phenotype.

### 3.2. Time-Lapse Analysis of Heterocyst Development in the pknH Mutant

**Figure 4:**
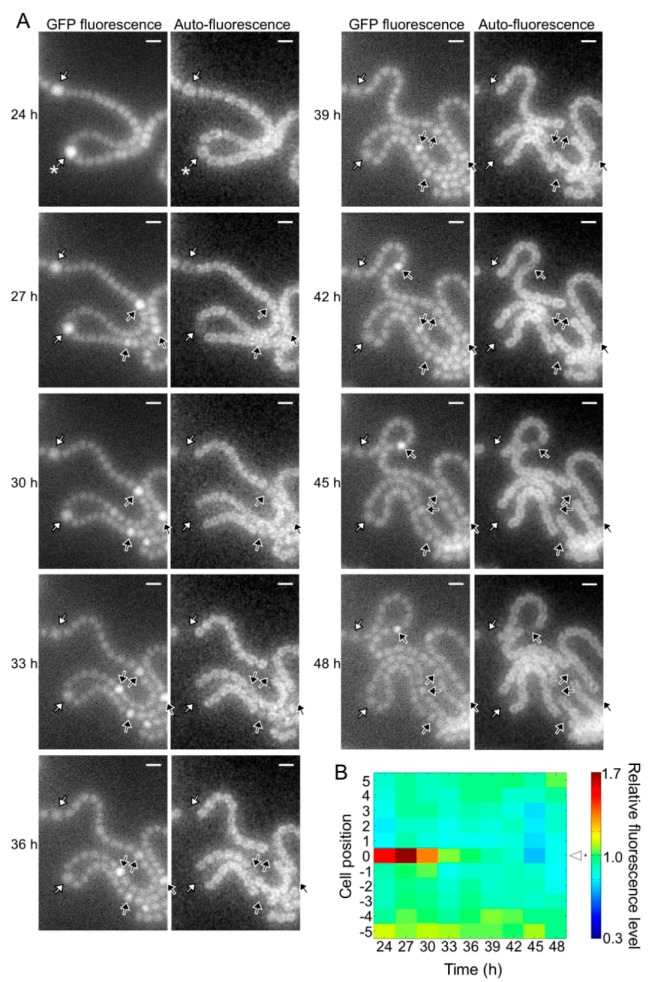
Spatiotemporal expression of the *hetR*-*gfp* transcriptional fusion gene in the wild type. (**A**) Left panels in each column are green fluorescent protein (GFP) fluorescence images and right panels are corresponding auto-fluorescence images. Sequential micrographs were taken at 3 h intervals from 24 to 48 h after nitrogen deprivation. White arrows, cells with high GFP fluorescence at 24 h; black arrows, high GFP fluorescence cells located in the middle of a string of vegetative cells. Scale bar, 5 μm. (**B**) Changes in the GFP fluorescence levels of heterocysts (cell position 0) and adjacent vegetative cells (cell positions 1 to 5 and −1 to −5). The fluorescence intensity of a heterocyst and five vegetative cells located on each side of the heterocyst was measured. We analyzed 30 groups of cells and a typical cell profile, including the heterocyst marked with an asterisk in the 24 h image of Figure 4A, is indicated.

**Figure 5:**
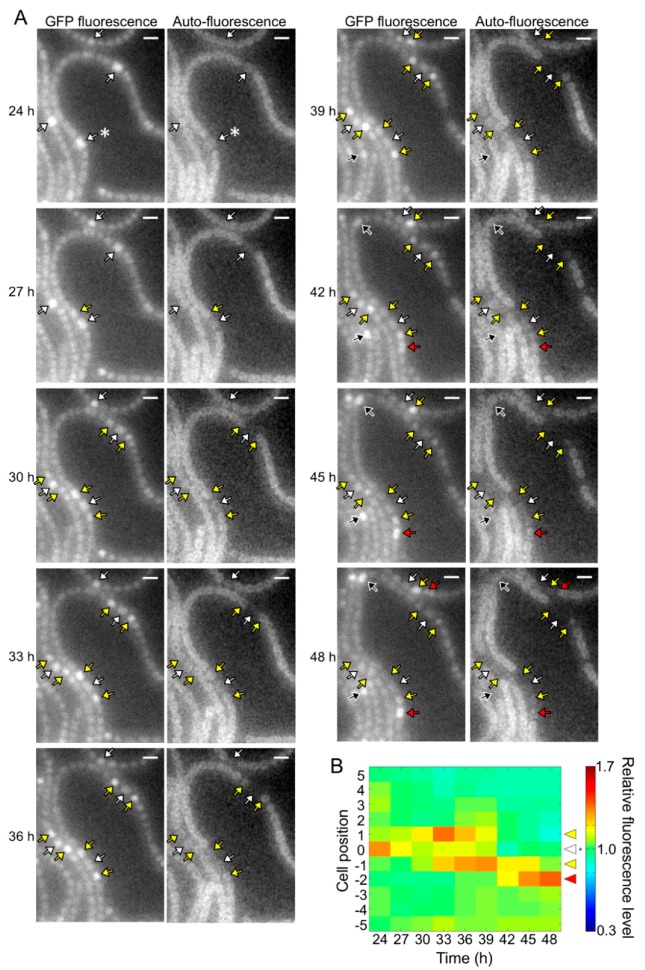
Spatiotemporal expression of the *hetR*-*gfp* transcriptional fusion gene in the *pknH* mutant. (**A**) Left panels in each column are GFP fluorescence images and right panels are corresponding auto-fluorescence images. White arrows, cells with high GFP fluorescence at 24 h; yellow and red arrows, high GFP fluorescence cells adjacent to the preexisting heterocysts; black arrows, high GFP fluorescence cells located in the middle of a string of vegetative cells. Scale bar, 5 μm. (**B**) Changes in the GFP fluorescence levels of heterocysts (cell position 0) and adjacent vegetative cells (cell positions 1 to 5 and −1 to −5). The fluorescence intensity of a heterocyst and five vegetative cells located on each side of the heterocyst was measured. We analyzed 30 groups of cells and a typical cell profile, including the heterocyst marked with an asterisk in the 24 h image of Figure 5A, is indicated.

The *pknH* mutant showed a normal heterocyst pattern 24 h after nitrogen deprivation, but Mchs were observed after 48 h (Figure 2). We conducted a time-lapse analysis of heterocyst development during the 24 to 48 h period. Heterocyst differentiation was induced by settling filaments on agar medium containing no combined nitrogen sources. Heterocyst formation was monitored by GFP fluorescence that was expressed from the *hetR* promoter and auto-fluorescence from photosynthetic pigments. At 24 h, high GFP fluorescence was observed in some cells and auto-fluorescence of these cells decreased during subsequent incubation, indicating that these cells were heterocysts (white arrows in Figure 4A). In the WT background, GFP fluorescence of cells located in the center of a string of vegetative cells increased at 27 h with the proliferation of vegetative cells, and then auto-fluorescence of these cells decreased (black arrows in Figure 4A). Thus, the heterocyst distribution was maintained from 24 to 48 h. Meanwhile, in the *pknH* mutant, GFP fluorescence increased in cells adjacent to preexisting heterocysts (yellow arrows in Figure 5A). These cells with high GFP fluorescence differentiated into heterocysts, as indicated by a decrease in auto-fluorescence, resulting in the formation of Mchs. Moreover, further differentiation was initiated at 42 h at a cell adjacent to Mchs (red arrows in Figure 5A). On the other hand, de novo differentiation of a single heterocyst was observed in the middle of a string of vegetative cells (black arrows in Figure 5A). Therefore, the Mch phenotype in the *pknH* mutant was not formed by simultaneous differentiation of contiguous vegetative cells but by sequential differentiation of vegetative cells adjacent to preexisting heterocysts. We quantified the GFP fluorescence level of each cell and analyzed the temporal changes in *hetR* expression in cells adjacent to heterocysts. In the WT background, an increase in *hetR* expression was not observed in cells adjacent to heterocysts (Figure 4B). However, in the *pknH* mutant, *hetR* expression was induced in cells adjacent to the preexisting heterocysts in 23 out of the 30 heterocysts analyzed (Figure 5B). These results suggest that vegetative cells adjacent to heterocysts would have a tendency to increase *hetR* expression and that PknH, which is exclusively expressed in heterocysts [16], suppresses the induction of *hetR* in neighboring vegetative cells.

## 4. Discussion

In the present study, we demonstrated that the *pknH* mutant exhibited a Mch phenotype by statically inducing heterocyst differentiation, indicating that *pknH* is involved in heterocyst pattern formation. We have previously shown that heterocysts of the *pknH* mutant are easily detached from the filaments and proposed that *pknH* is involved in maintaining the connection between heterocysts and vegetative cells [16]. However, detachment of the heterocysts from the filaments in the *pknH* mutant would be initiated by breaking the connection between contiguous heterocysts, not by breaking the connection between heterocysts and vegetative cells. Heterocysts have a thick envelop outside the cell wall and are connected to adjacent cells by the narrowed septum at their poles [25]. The fragile connection between contiguous heterocysts would be responsible for fragmentation of the filaments in the *pknH* mutant, although the possibility that differences in culture conditions, such as bubbling with air containing 1% CO_2_ in the previous study or incubation on an agar medium containing 5 mM NaHCO_3_ under atmospheric CO_2_ levels in this study, affect the phenotype of the *pknH* mutant cannot be ruled out.

The *pknH* mutant initially exhibited the normal heterocyst pattern, but 48 h after nitrogen deprivation, one-half of the heterocysts were contiguous to another heterocyst (Figure 2). Thus, *pknH* is necessary for maintaining the heterocyst pattern during diazotrophic growth. Time-lapse analysis indicated that the expression of *hetR* increased in vegetative cells adjacent to preexisting heterocysts in the *pknH* mutant (Figure 5). Suppression of heterocyst differentiation of vegetative cells adjacent to preexisting heterocysts is likely to be stronger than that of vegetative cells separated from heterocysts because of the nitrogen-fixing products supplied by the heterocysts. During heterocyst differentiation, the expression of *hetR* is initially upregulated in groups of filament cells and then becomes restricted to a single cell [26,27]. An elevated signal that upregulates the expression of *hetR* might be maintained in cells adjacent to heterocysts.

The molecular mechanisms by which PknH suppresses *hetR* expression in cells adjacent to heterocysts, where the *pknH* gene is not expressed, remains to be elucidated; however, it was shown that Asp-184 of PknH, which is conserved in the catalytic domain of Ser/Thr kinases, was essential for suppression of Mch (Figure 2). The phosphorylation activity of PknH could be related to the *hetN*-dependent downregulation of HetR because the delayed Mch phenotype was also observed in the *hetN* mutant [13]. Since the *hetN* transcript levels were not affected by *pknH* disruption (Figure 3), PknH would not be involved in the transcriptional regulation of *hetN*. The *hetN*-dependent signal is transferred to adjacent vegetative cells [28], and the septal protein SepJ and predicted ABC transporter HetC play important roles in the intercellular transfer of the *hetN*-dependent signal [29,30]. PknH could enhance the intercellular movement of the *hetN*-dependent signal through interaction with these proteins. Alternatively, PknH might be related to processing the HetN protein. It has been reported that there were PatS-processing activities within cell extracts of *Anabaena* [31]. *patS* encodes a protein with 17 amino acids [11], but the PatS C-terminal hexapeptide (ERGSGR) has been proposed to be an active form of PatS [9]. Because the full-length HetN peptide is not intercellularly transferred, HetN should be cleaved before it is transferred [28]. HetN-processing activities might be activated by PknH. The *pknH*/*hetN* double mutant would be useful for understanding the relationship between PknH and HetN. Moreover, identification of proteins that are phosphorylated by PknH is crucial for elucidation of PknH’s function in maintaining the heterocyst pattern.

## Figures and Tables

**Table 1:** Primers used in this study.

Primer	Sequence (5′-3′)
1336-5F	ATGAGCTCTTTACTGGTTGCCTGCTGTG
PknH-R	AAGTCGACGCATCACCGACACATAAAC
RTrrn16S-F2	GCAAGTCGAACGGTCTCTTC
RTrrn16S-R2	GGTATTAGCCACCGTTTCCA
RThetN-F	CATGATGGAACGCGGTAGTG
RThetN-R	AATTCCTGACGCATCGCATC
